# The impact of driver mutation on the treatment outcome of early-stage lung cancer patients receiving neoadjuvant immunotherapy and chemotherapy

**DOI:** 10.1038/s41598-022-07423-w

**Published:** 2022-02-28

**Authors:** Po-Lan Su, Jung-Yueh Chen, Chang-Yao Chu, Yi-Lin Chen, Wan-Li Chen, Kuan-Yu Lin, Chung-Liang Ho, Jeng-Shiuan Tsai, Szu-Chun Yang, Chian-Wei Chen, Yi-Lin Wu, Yau-Lin Tseng, Chao-Chun Chang, Yi-Ting Yen, Chia-Ying Lin, Chien-Chung Lin, Wu-Chou Su

**Affiliations:** 1 Department of Internal Medicine, National Cheng Kung University Hospital, College of Medicine, National Cheng Kung University, Tainan, Taiwan; 2 Department of Internal Medicine, E-Da Hospital, Kaohsiung, Taiwan; 3 School of Medicine, I-Shou University, Kaohsiung, Taiwan; 4 Department of Pathology, Chi-Mei Medical Center, Tainan, Taiwan; 5 Department of Pathology, National Cheng Kung University Hospital, College of Medicine, National Cheng Kung University, Tainan, Taiwan; 6 Department of Nursing, National Cheng Kung University Hospital, College of Medicine, National Cheng Kung University, Tainan, Taiwan; 7 Department of Surgery, National Cheng Kung University Hospital, College of Medicine, National Cheng Kung University, Tainan, Taiwan; 8 Department of Medical Imaging, National Cheng Kung University Hospital, College of Medicine, National Cheng Kung University, Tainan, Taiwan; 9 Institute of Clinical Medicine, National Cheng Kung University Hospital, College of Medicine, National Cheng Kung University, Tainan, Taiwan; 10 Department of Biochemistry and Molecular Biology, College of Medicine, National Cheng Kung University, Tainan, Taiwan; 11 Department of Oncology, National Cheng Kung University Hospital, College of Medicine, National Cheng Kung University, Tainan, Taiwan; 12 Center of Applied Nanomedicine, National Cheng Kung University, Tainan, Taiwan

**Keywords:** Non-small-cell lung cancer, Cancer immunotherapy

## Abstract

Neoadjuvant immunotherapy and chemotherapy have improved the major pathological response (MPR) in patients with early-stage operable non-small cell lung cancer (NSCLC). This study aimed to assess whether the presence of targetable driver mutations affects the efficacy of the combination of immunotherapy and chemotherapy. We enrolled patients with early-stage operable NSCLC who received preoperative neoadjuvant therapy between January 1, 2017, and December 30, 2020. Neoadjuvant therapy was delivered with platinum-doublet chemotherapy; moreover, pembrolizumab was added at the attending physician’s discretion based on patient’s request. Pathological responses were assessed; moreover, disease-free survival was estimated. Next-generation sequencing was performed in case sufficient preoperative biopsy specimens were obtained. We included 23 patients; among them, 11 received a combination of neoadjuvant immunotherapy and chemotherapy while 12 received neoadjuvant chemotherapy alone. The MPR and pathological complete response rates were 54.5% and 27.3%, respectively, in patients who received a combination of neoadjuvant immunotherapy and chemotherapy. These rates were significantly higher than those in patients who only received neoadjuvant chemotherapy. Three patients in the combination group experienced disease recurrence during the follow-up period even though two of them showed an MPR. These three patients had targetable driver mutations, including an EGFR exon 20 insertion, EGFR exon 21 L858R substitution, and MET exon 14 skipping. Only one patient who remained disease-free had a targetable driver mutation. Among patients with early-stage operable NSCLC requiring neoadjuvant therapy, comprehensive genomic profiling is crucial before the administration of the combination of neoadjuvant immunotherapy and chemotherapy.

## Introduction

Tumor relapse in patients with stage II and III non-small cell lung cancer (NSCLC) remains a great challenge. The 5-year survival rates in patients with N1- and N2-positive NSCLC are 49% and 36%, respectively^1^. Although neoadjuvant chemotherapy significantly improves the overall survival in patients with operable NSCLC, the absolute benefit was reported only to be 6% in the 5-year overall survival rate^2^. There is a need for novel innovative drug combination strategies to improve the postoperative overall survival. Immunotherapy has improved the overall survival in patients with advanced NSCLC. Phase 3 studies have demonstrated that pembrolizumab or atezolizumab significantly improve overall survival among patients with high programmed death-ligand 1 (PD-L1) expression^3,4^. Combining immunotherapy and chemotherapy has further improved the overall survival of patients with stage IV NSCLC^5,6^. Given the success of immune checkpoint inhibitors in advanced NSCLC, there is growing evidence regarding their use in patients with early-stage operable NSCLC.

Forde et al. reported that neoadjuvant nivolumab allowed a major pathological response (MPR) rate of 45% in patients with stage II to III operable NSCLC^7^. However, a subsequent larger phase II LCMC3 study reported that neoadjuvant atezolizumab for untreated stage IB–IIIB operable NSCLC without EGFR or ALK mutations allowed an MPR rate of only 20% (30/147; 95% CI: 14–28%) and a pathological complete response (pCR) rate of only 7% (10/147; 95% CI: 3–12%)^8^. A single arm cohort study also revealed that neoadjuvant pembrolizumab provides MPR of only 27% and pCR of only 13.3%^9^. To improve the pathological response, there is increasing evidence regarding the surgical outcomes through the combination of neoadjuvant immunotherapy and chemotherapy. A phase 2 single-arm multicenter NADIM study on the treatment efficacy of neoadjuvant nivolumab and chemotherapy reported that 83% and 63% of the patients had MPR and pCR, respectively^10^. Moreover, a multicenter single-arm phase 2 study on neoadjuvant atezolizumab and chemotherapy found that 57% and 33% of the patients had MPR and pCR, respectively^11^. The recent phase 3 checkmate 816 study observed MPR and pCR in 46.8% and 30.5%, respectively, in patients who received neoadjuvant nivolumab and chemotherapy followed by surgery^12^. Current ongoing phase 3 trials also evaluated the role of different immunotherapy and chemotherapy combination in neoadjuvant therapy (ClinicalTrials.gov identifier: NCT03425643, NCT03456063, and NCT03800134). However, the wide variation of MPR rates indicates the need for studies to determine a predictive biomarker.

There remains controversial evidence regarding predictive biomarkers. Studies on the PD-L1 expression level as a predictive biomarker have reported inconsistent results^10–12^. Additionally, the studies using tumor mutation burden^7^ or circulating tumor DNA^12^ as predictive biomarkers were also limited. This study aimed to analyze the treatment outcomes of the combination of neoadjuvant pembrolizumab and chemotherapy, as well as the association of treatment efficacy with predictive biomarkers, including PD-L1 expression level and genomic alterations.

## Material and method

### Patient population

We retrospectively reviewed patients who diagnosed with stage I to III NSCLC at a tertiary hospital from January 1, 2017, to December 30, 2020. All patients had undergone Chest computed tomography (CT) scans, whole-body bone scans, and brain imaging (CT or magnetic resonance imaging) before treatment. Then, the patients were staged based on the tumor, node, metastasis (TNM) system proposed by the American Joint Committee on Cancer, 7th edition. Baseline characteristics, including age, sex, tumor histology, and TNM stage, were recorded. Transbronchial lymph node aspiration or positron emission tomography was performed to define the lymph node stage. After multidisciplinary team discussion, neoadjuvant chemotherapy was recommended for a proportion of patients, except for patients with unresectable disease, poor performance status, or relatively limited disease which did not require extensive surgical resection. The multidisciplinary team consisted of various specialists, including chest physicians, thoracic surgeons, medical oncologists, radiation oncologists, radiologists, nuclear medicine physicians, and pathologists. The treatment strategy of patients with complicated condition or stage III tumor were discussed at the weekly meeting. Neoadjuvant chemotherapy was administered using a 4-cycle platinum-doublet regimen (cisplatin, carboplatin, and docetaxel). Moreover, two doses of pembrolizumab 100 mg were administered at the attending physician’s discretion based on patient’s request, instead of a pre-selected patient group. We then perform retrospective analysis of treatment efficacy among patients who received neoadjuvant chemotherapy. The informed consent was obtained from all participants. This study was reviewed and approved by the Review Board and Ethics Committee of the National Cheng Kung University Hospital (NCKUH B-ER-109-182). All data were anonymized following the approved guidelines and the Declaration of Helsinki.

### Tumor response and survival analysis

The tumor response to neoadjuvant therapy was evaluated using radiologic and pathological criteria. The chest CT scan was arranged after completing neoadjuvant therapy to determine the radiological response and at subsequent 12-week intervals after surgery until disease recurrence. Disease-free survival was calculated from the date of surgery until the date of radiological progression based on the Response Evaluation Criteria in Solid Tumors v1.1^13^ or death, with censoring at the date of the last follow-up if the patient did not show recurrence. The percentage of residual viable tumors, necrosis, and stroma in post-operative specimens was quantified by a pathologist as per the IASLC multidisciplinary recommendations^14^. We used the average value of the percentage of residual tumor to determine the pathological response as described by Junker et al.^15^ and IASLC recommendation^14^. As proposed by Junker et al., the regression status was classified into four categories as follows: score I (no tumor regression), score IIa (tumor regression with ≥ 10% viable tumors), score IIb (tumor regression with < 10% viable tumors), and score III (no viable tumors in both primary tumors and regional lymph nodes). The classification proposed by the IASLC includes two categories, with MPR being defined as < 10% residual tumor and pCR being defined as no viable tumor discovered^16^.

To determine the predictive factors for pathological response, a lung pathologist assessed the preoperative and postoperative tumor specimens following established recommendations of the World Health Organization^17^. The PD-L1 expression level, which was presented as a tumor proportion score, was assessed using the Dako 22C3 pharmDx system (Agilent Technologies Inc., Santa Clara, CA, USA) assay^18^. Additionally, in case sufficient biopsy specimens were obtained, the targetable driver mutation was assessed through next-generation sequencing using the *QIAGEN GeneReader* NGS system and QIAact *Lung All-in-One assay*.

### Statistical analysis

Categorical variables were compared using the chi-square test or Fisher’s exact test. Continuous variables were compared using Student’s t-test or the Wilcoxon rank-sum test. Statistical analysis was conducted using SAS version 9.4 (SAS Institute Inc., Cary, NC, USA). All reported *P values* were two‐sided.

## Results

### Patients

**Figure 1:**
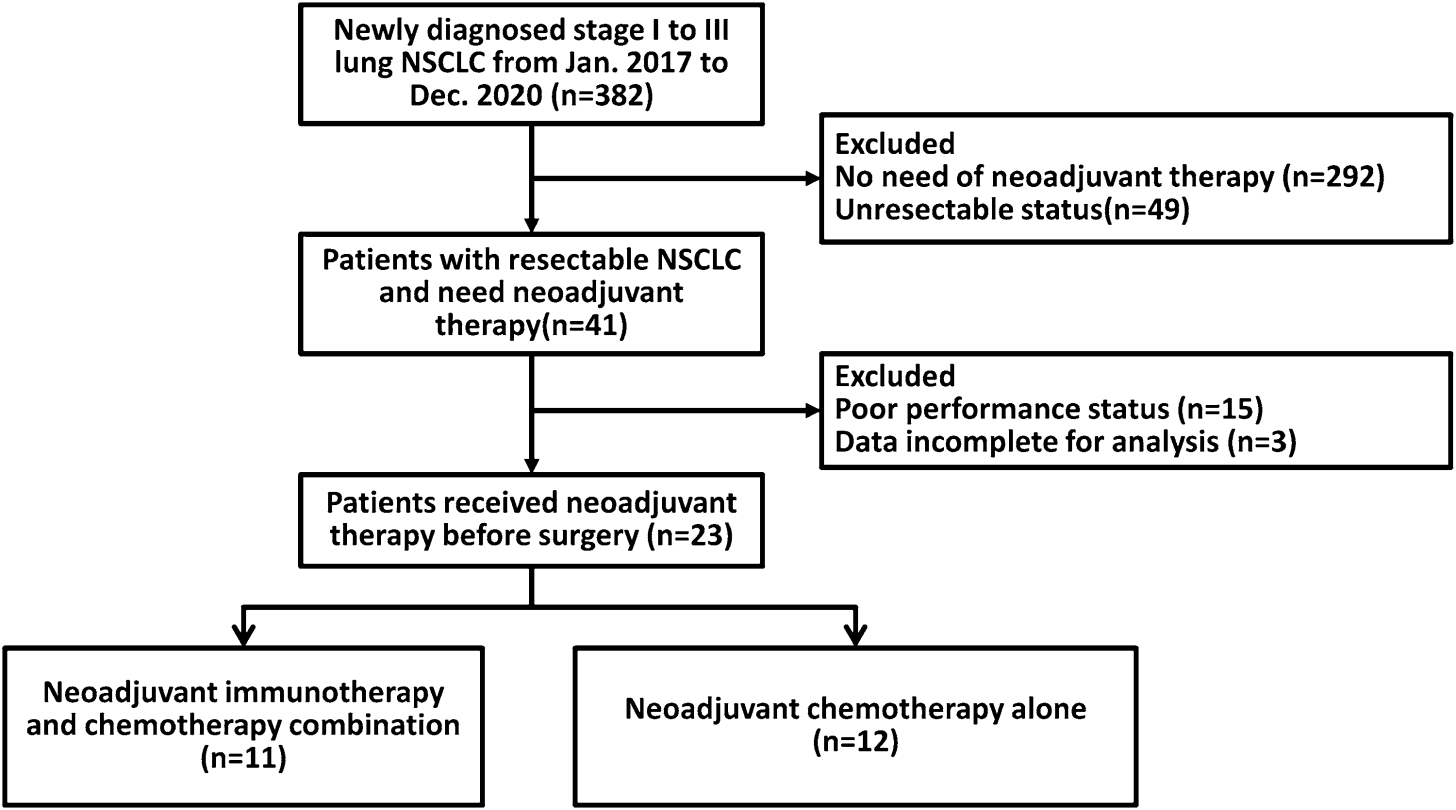
Flow chart describing the enrollment of patients.

A total of 23 patients were enrolled, including 11 and 12 patients received neoadjuvant pembrolizumab and chemotherapy (combination group) and neoadjuvant chemotherapy only (chemotherapy group), respectively. Figure 1 presents a detailed flowchart for enrolling participants. Table 1 summarizes the baseline characteristics of the patients. The median age was 63.8 years; moreover, 18 of the participants were male. The histological subtypes included adenocarcinoma (n = 11) and squamous cell carcinoma (n = 12). Further, 21 patients had stage III disease; further, two patients had stage II disease but required neoadjuvant therapy. There was no difference in baseline characteristics between the combination and chemotherapy group, including age, sex, histological subtype, tumor size, nodal status, and stage distribution (Table 1).

**Table 1:** Baseline characteristics.

	Total population (N = 23)	Combined chemotherapy and immunotherapy (N = 11)	Chemotherapy alone (N = 12)	P value
**Age, median (years)**	63.8	61.0	64.0	0.554
≥ 60	16	7	9	
< 60	7	4	3	
**Sex, n (%)**				0.692
Male	18	9	9	
Female	5	2	3	
**Histology**				0.827
Adenocarcinoma	11	5	6	
Squamous cell carcinoma	12	6	6	
**Tumor size**				0.286
≥ 3 cm	22	10	12	
< 3 cm	1	1	0	
**Nodal involvement, n (%)**				0.538
N1	5	3	2	
N2	18	8	10	
**Stage, n (%)**				0.799
II	2	1	1	
IIIA	10	4	6	
IIIB	11	6	5	

### Tumor response and survival analysis

**Figure 2:**
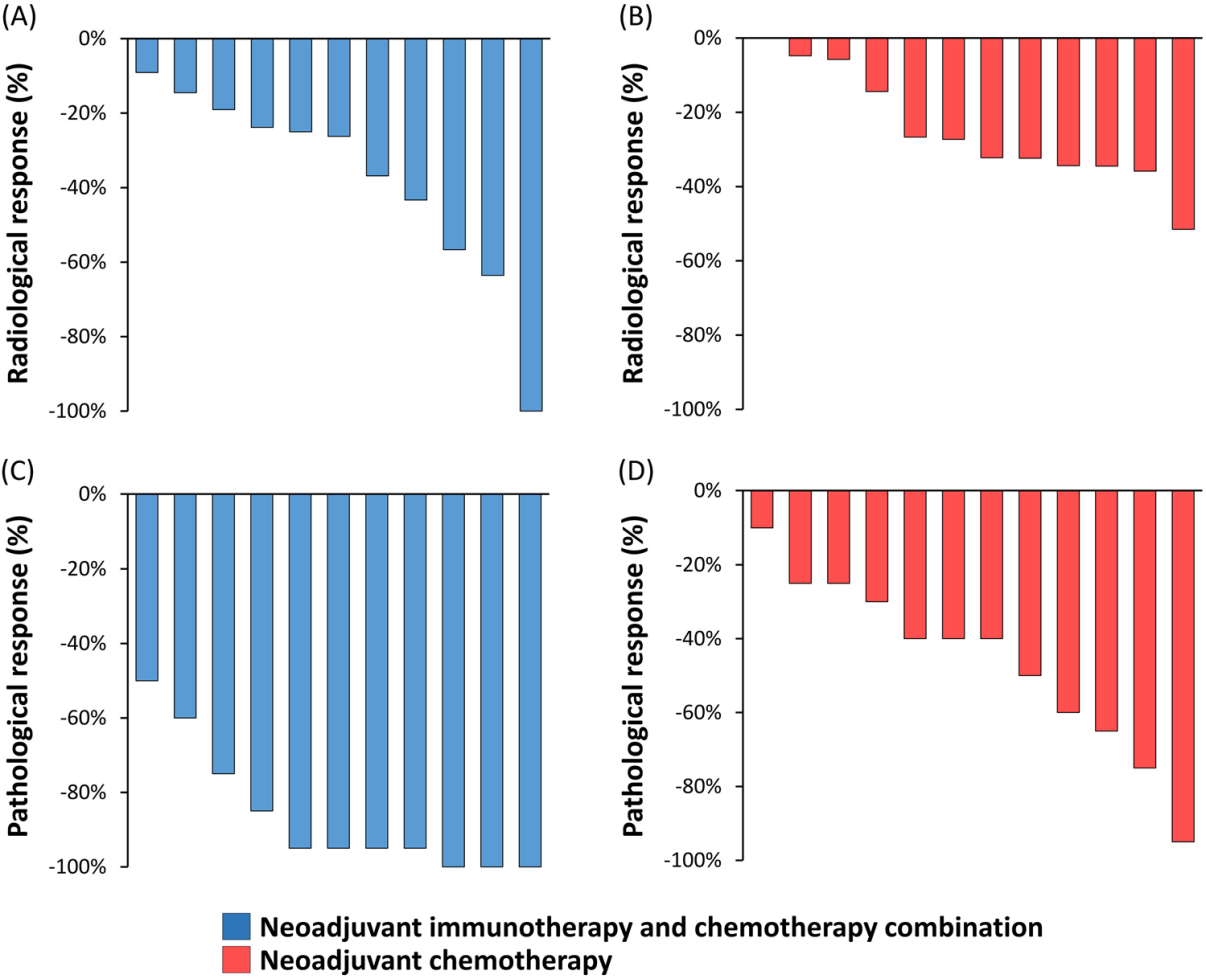
The radiological response in patients who received the combination of neoadjuvant immunotherapy and chemotherapy **(A)** and in those who received neoadjuvant chemotherapy alone **(B)**. The pathological response in patients who received the combination of neoadjuvant immunotherapy and chemotherapy **(C)** and in those who received neoadjuvant chemotherapy alone **(D)**.

The median follow-up period was 18.3 months. The radiological objective response rate (ORR) in the combination and chemotherapy groups were 45.5% (Fig. 2A) and 58.3%, (Fig. 2B), respectively (P = 0.537). Similarly, the disease control rates were 100% in both groups. For pathological assessment, the combination group had a significantly higher MPR rate (63.6%, Fig. 2C) than the chemotherapy group (8.3%, Fig. 2D) (P = 0.005). Furthermore, the combination group had a significantly higher pCR (27.3%, Fig. 2C) than the chemotherapy group (0%, Fig. 2D) (P = 0.052). Table 2 details the tumor response and pathological assessment of the combination group. All patients with an MPR had pathological nodal clearing (postoperative N0 status, Table 2). There was no significant correlation between the radiological response and pathological response. The immunohistochemical staining of PD-L1 and the H&E stain of pathological response were illustrated in supplementary material Figs. S1 and S2.

**Table 2:** Pathological response of patients who received neoadjuvant chemotherapy and immunotherapy.

ID	Histology	PD-L1 (%)		Tumor size (cm)		Nodal status		Pathological response	Junker Regression	Pathological assessment according to IASLC (%)			DFS (months)	Recurrence
		Biopsy	Surgery	Biopsy	Surgery	Biopsy	Surgery			Tumor	Necrosis	Stroma		
1	Squamous	95	90	9.6	3.5	2	0	PR	IIa	50	0	50	22.30	No
2	Adenocarcinoma	0	100	6.1	4.5	1	0	PR	IIa	40	10	50	9.31	No
3	Adenocarcinoma	90	90	3.8	2.4	2	2	PR	IIa	25	0	75	12.13	Yes
4	Squamous	75	95	6.3	4.8	2	0	PR	IIa	15	50	35	17.48	No
5	Squamous	0	100	5	5.3	1	0	MPR	IIb	5	65	30	18.16	No
6	Adenocarcinoma	90	100	3.2	2.4	2	0	MPR	IIb	5	0	95	15.54	Yes
7	Adenocarcinoma	20	70	6	3.4	2	0	MPR	IIb	5	35	60	9.31	No
8	Adenocarcinoma	90	70	6.6	6	2	0	MPR	IIb	5	20	75	9.25	Yes
9	Squamous	60	NA^a^	3	1.3	1	0	pCR	III	0	0	100	1.67	No
10	Squamous	NA^a^	NA^a^	4.3	0	2	0	pCR	III	0	0	100	30.20	No
11	Squamous	20	NA^a^	2.1	1.7	2	0	pCR	III	0	20	80	14.66	No

*MPR* major pathological response, *pCR* pathological complete response, *PR* partial response.

^a^Not available because of insufficient tissue.

**Figure 3:**
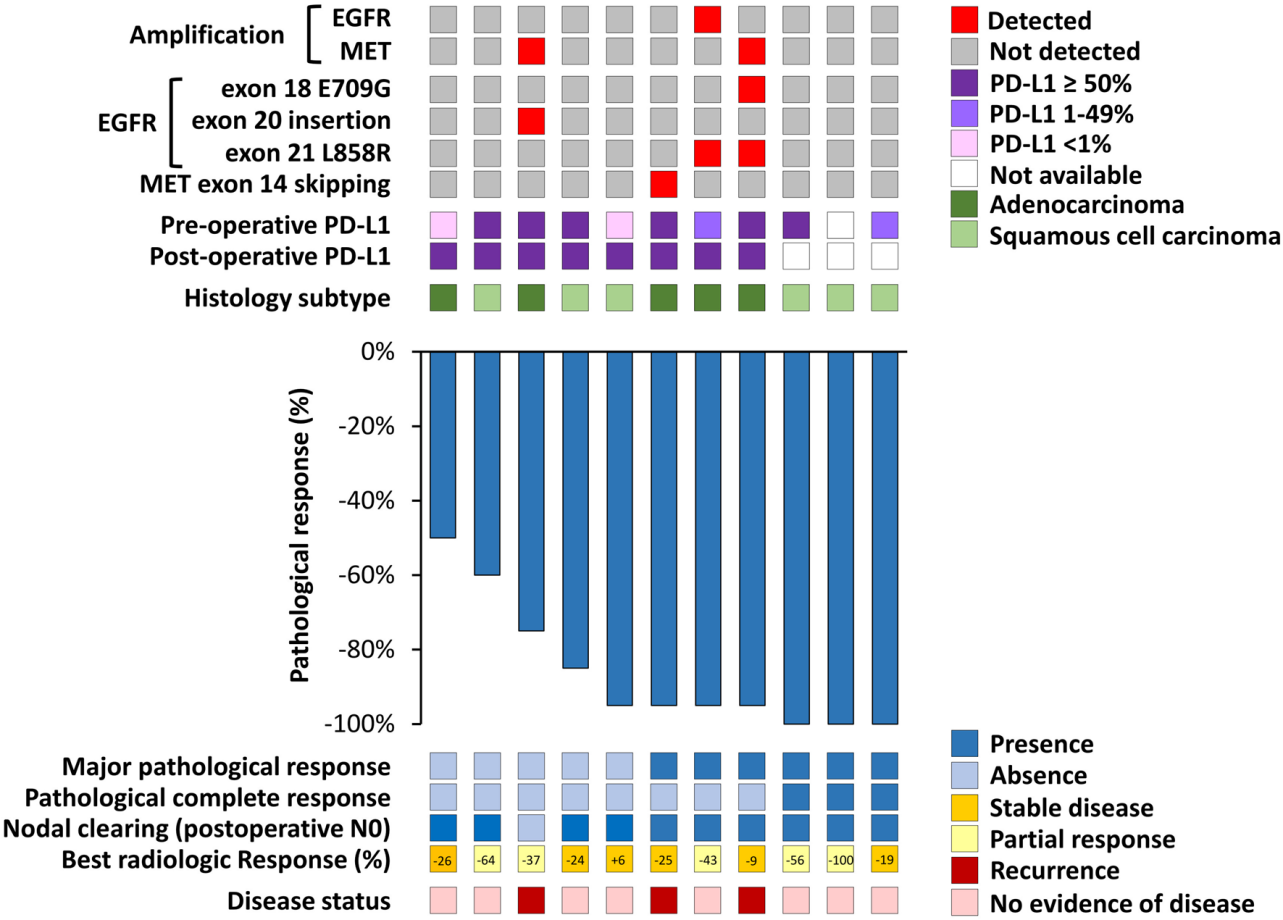
The correlation between pathological response and individual patient characteristics, including genomic data, histological subtype, PD-L1 expression, and disease status, in patients who received the combination of neoadjuvant immunotherapy and chemotherapy.

Figure 3 summarizes the relationship between pathological response and individual patient characteristics, including genomic alterations, histological subtypes, PD-L1 expression level, and disease status. Neither the PD-L1 expression level nor the histological subtype could predict the MPR. During the follow-up period, three patients in the combination group experienced disease recurrence. All three patients had high PD-L1 expression in preoperative biopsy specimens; among them, two patients had an MPR in postoperative assessment. After next-generation sequencing, all three patients had targetable driver mutations, including an EGFR exon 20 insertion, complex EGFR mutation (exon 21 L858R substitution and exon 18 E709G substitution), and MET exon 14 skipping (splice donor site mutation). Further, two of these patients harbored MET amplification, with copy number gains of 2.89 and 3.94, respectively. Contrastingly, there was a significantly lower frequency of targetable driver mutations among the remaining eight patients with a disease-free status during the follow-up period, with only one patient having a targetable driver mutation (EGFR exon 21 L858R substitution) and EGFR amplification (copy number gain 10.23) (P = 0.007). The detailed genomic sequencing data of individual patients were listed in supplementary material Fig. S3.

## Discussion

Our findings demonstrated that neoadjuvant pembrolizumab and chemotherapy provided better MPR and pCR compared with neoadjuvant chemotherapy, which was consistent with previous findings^10–12^. The pCR rate in the combination group was compatible with findings of the recent phase 3 study Checkmate 816, which demonstrated that the combination of neoadjuvant nivolumab and chemotherapy allowed a pCR rate of 24.0%. Moreover, all our patients with pCR presented with nodal clearing (downstaging from N2 to N0)^12^. Consistent with the aforementioned studies, we found that the combination of neoadjuvant immunotherapy and chemotherapy could be the mainstay treatment modality in patients with operable early-stage NSCLC requiring neoadjuvant therapy.

Numerous preclinical studies have provided evidence that neoadjuvant immunotherapy can provide better survival for patients with resectable tumors compared with administration of the same therapy in adjuvant settings. Immune checkpoint inhibitors can activate suppressed intratumoral cytotoxic T cells, which mediate antitumor immune responses, as well as promote interactions between cytotoxic T cells and antigen‐presenting cells^19^. A preclinical study regarding the efficacy of various immunotherapies (anti-CD25, anti-PD-1, or combined anti-PD-1 and anti-CD137) reported that neoadjuvant therapy had improved long-term survival over adjuvant therapy in murine models of triple‐negative breast cancer^20^. Pathological assessment of the resected tumor revealed an increased density of intratumoral cytotoxic T cells, which may be associated with improved treatment efficacy^20^. However, a clinical study demonstrated suboptimal MPR and pCR with neoadjuvant immunotherapy^8^. There is a need for further research regarding new combinations for neoadjuvant therapy.

Preclinical findings have demonstrated that combining chemotherapy and immunotherapy may provide better survival benefits. This combination could inhibit tumor growth and stimulate immune reactions to tumor cells. In addition to increasing the release of tumor neoantigens to augment immunogenicity, chemotherapy inhibits regulatory T cells and enhances the intratumoral activity of cytotoxic T cells^21,22^. Chemotherapy could block the signaling of signal transducer and activator of transcription 6, enhance the effector immune response by modulating the expression of PD-L1 and mannose-6-phosphate receptor, and stimulate immunogenic cell death through the production of adenosine triphosphate and high-mobility group protein box-1^23,24^. Additionally, the phase 3 Checkmate816 study observed better pCR in patients receiving the combination of neoadjuvant nivolumab and chemotherapy^12^. Taken together, the combination of neoadjuvant immunotherapy and chemotherapy should be the mainstay strategy for patients with early-stage NSCLC.

However, biomarkers for predicting patients who could benefit from combination therapy remain unclear. A previous study on the efficacy of immunotherapy and chemotherapy in advanced NSCLC reported similar hazard ratios of overall survival were similar among patients with different PD-L1 expression levels^5,6^. However, patients with PD-L1 expression levels > 50% had the longest overall survival when they received the combination of immunotherapy and chemotherapy^5,6^. The phase 2 NADIM study, which used nivolumab and chemotherapy as neoadjuvant therapy, reported that PD-L1 expression levels > 25% could predict MPR with 65% sensitivity and 100% specificity^10^. However, in the Checkmate 816 study, both patients with positive and negative PD-L1 expression benefited from neoadjuvant nivolumab combined with chemotherapy^12^. Further, the phase 2 study using neoadjuvant atezolizumab and chemotherapy reported that the MPR was not dependent on the PD-L1 expression level^11^. In our study, the PD-L1 expression level varied widely across both patients with and without MPR. Future prospective studies should assess the association of the PD-L1 expression level with pathological response.

Another unfavorable prognostic factor of immunotherapies in patients with advanced NSCLC is the presence of EGFR or ALK mutations. A meta-analysis demonstrated that using immune checkpoint inhibitors did not benefit the overall survival of patients with EGFR mutations^25^. However, it remains unclear whether targetable driver mutations decrease the efficacy of the combination of neoadjuvant immunotherapy and chemotherapy. Previous studies on the combination of neoadjuvant immunotherapy and chemotherapy have employed different patient enrollment strategies. The NADIM study^10^ excluded patients with EGFR or ALK mutations; contrastingly, the phase 2 study^11^ using atezolizumab enrolled all patients with early-stage lung cancer. Although the MPR rate was similar across these studies, they did not perform disease-free survival analysis in the subgroup of patients with targetable driver mutations^9,11^. In our study, three of the four patients with targetable driver mutations had MPR after receiving the combination of neoadjuvant immunotherapy and chemotherapy. However, all three patients who experienced disease recurrence had targetable driver mutations, including an EGFR exon 20 insertion, EGFR exon 21 L858R substitution, and MET exon 14 skipping. To our knowledge, this is the first study to demonstrate that an EGFR exon 20 insertion or MET exon 14 skipping can shorten the disease-free survival after administration of the combination of neoadjuvant immunotherapy and chemotherapy. Future studies should investigate better neoadjuvant therapy for patients with targetable driver mutations.

There is growing evidence regarding the use of neoadjuvant EGFR-tyrosine kinase inhibitors (TKIs) in patients with early-stage operable EGFR-mutant NSCLC^26^. Earlier phase 2 single-arm studies using EGFR-TKI as neoadjuvant therapy revealed ideal ORR, MPR, and disease-free survival^27,28^. Another phase 2 study reported that patients receiving neoadjuvant erlotinib had a higher MPR rate and significantly longer disease-free survival than those receiving neoadjuvant chemotherapy^29^, which suggests that neoadjuvant targeted therapy may be a better strategy. Given the improved survival allowed by combined chemotherapy and gefitinib in patients with advanced-stage EGFR-mutant NSCLC^30,31^, the ongoing phase 3 NEOADAURA study is evaluating the treatment efficacy of the combination of neoadjuvant osimertinib and chemotherapy^32^. Additionally, the LCMC4 trial (ClinicalTrials.gov identifier: NCT04712877) will further validate the role of comprehensive genomic profiling and neoadjuvant targeted therapy in patients with early-stage operable NSCLC. Neoadjuvant targeted therapy might be an alternative for patients with targetable driver mutations.

There are several possible explanations regarding the decreased efficacy of immune checkpoint inhibitors in patients with targetable driver mutations. Previous studies reported that patients with EGFR, ALK, ROS1, and RET mutations carry relatively low tumor mutation burden (TMB) (3–6 mutations/Mb, 2–4 mutations/Mb, 4 mutations/Mb, and 4.8 mutations/Mb, respectively)^33,34^. Another study reported that > 50% and ≈ 10% of patients with EGFR mutations had a PD-L1 expression level < 1% and > 50%, respectively^35^. The low TMB and PD-L1 expression levels might be associated with a low response rate and short progression-free survival with the use of PD-1 inhibitors^36^. Additionally, high PD-L1 expression levels may be secondary to the signaling activity of the EGFR pathway, including the IL-6/JAK/STAT3 and p-ERK1/2/p-c-Jun pathways, rather than being the interacting molecule with intratumoral immune cells^37^. Moreover, the presence of targetable driver mutations is associated with an immunosuppressive tumor environment. For example, an EGFR mutation is associated with a decreased density of intratumoral cytotoxic T cells^38^; moreover, it results in immune escape by overactivation of regulatory T cells via the EGFR/GSK-3β/Foxp3 pathway^39^. Additionally, the EGFR-mutant cancer cells secrete colony stimulating factor 1, which converts tumor-associated macrophages into the M2 phenotype^40^. The low TMB/PD-L1 levels and immunosuppressive tumor microenvironment result in poor response to immune checkpoint inhibitors. Taken together, the presence of targetable driver mutations might be associated with early recurrence in patients receiving the combination of neoadjuvant immunotherapy and chemotherapy.

This study has some limitations. First, this was a single-center small-sample sized study. However, the baseline characteristics were similar between the combination and chemotherapy groups. In addition, because patients with poor performance status were not recommended to received neoadjuvant therapy by multidisciplinary team (Fig. 1), all patients in both groups had good performance status. Moreover, the MPR and pCR rates were similar to those reported in the phase 3 Checkmate 816 study for patients who received the combination of neoadjuvant nivolumab and chemotherapy. Our participants may represent the general patient population. Second, although patients with MPR showed varying PD-L1 expression levels, which implies that PD-L1 could not predict the MPR rate, the sample size was limited. Nonetheless, previous findings regarding the role of PD-L1 in predicting the MPR rate remain inconsistent^9–12^. Moreover, the MPR rate may not be associated with disease-free survival in patients with targetable driver genes. There is a need for future prospective studies to validate the predictive biomarkers for the combination of neoadjuvant immunotherapy and chemotherapy. Third, the genomic tests were retrospectively assessed and only four patients in present study had targetable driver mutations, which precludes definitive conclusions regarding the role of targetable driver mutations in patients who received the combination of neoadjuvant immunotherapy and chemotherapy. Despite the limitation in patient number, our study highlighted the potential deteriorated effect of the targetable driver mutations in combined neoadjuvant immunotherapy and chemotherapy. Moreover, data regarding the role of targetable driver genes in disease-free survival remain unclear. Furthermore, one patient who suffered from disease progression after the combination of neoadjuvant immunotherapy and chemotherapy followed by surgery had MET exon 14 skipping. This genetic alteration has not been reported as a poor prognostic factor for the combination of immunotherapy and chemotherapy before. Forth, one of four patients with targetable driver mutations remained in disease-free status, which implied that the immunotherapy and chemotherapy combination might provide benefit in certain group of patients with targetable driver mutations. In a phase 2 study regarding the efficacy of neoadjuvant atezolizumab and chemotherapy, two of four patients with EGFR mutation had pCR^11^. However, in the study, disease-free survival in that patient subgroup was not analyzed. Given the nature of decreased immune cell infiltration in NSCLC with targetable driver mutation, whether the combination of immunotherapy could provide survival benefit warrant more clinical studies^41^. Moreover, the ongoing LCMC4 study (ClinicalTrials.gov identifier: NCT03515837) and phase 3 NEOADAURA will provide the data regarding neoadjuvant targeted therapy, which could provide more information about the choice of neoadjuvant therapy in patients with early stage NSCLC and targetable driver mutations.

In summary, among patients with operable stage II to IIIA NSCLC preoperatively receiving the combination of neoadjuvant immunotherapy and chemotherapy, the presence of targetable driver mutations may be associated with early recurrence. Therefore, comprehensive genomic profiling might have important role in patients with early-stage NSCLC. Future prospective studies are warranted to validate our findings.

## Supplementary Information

Supplementary Figure S1.

Supplementary Figure S2.

Supplementary Figure S3.

Supplementary Legends.
