# Supplementary figures and images for: The impact of driver mutation on the treatment outcome of early-stage lung cancer patients receiving neoadjuvant immunotherapy and chemotherapy
Source: Sci Rep. 2022 Feb 28;12:3319. doi: 10.1038/s41598-022-07423-w (PMC8885645; doi:10.1038/s41598-022-07423-w)

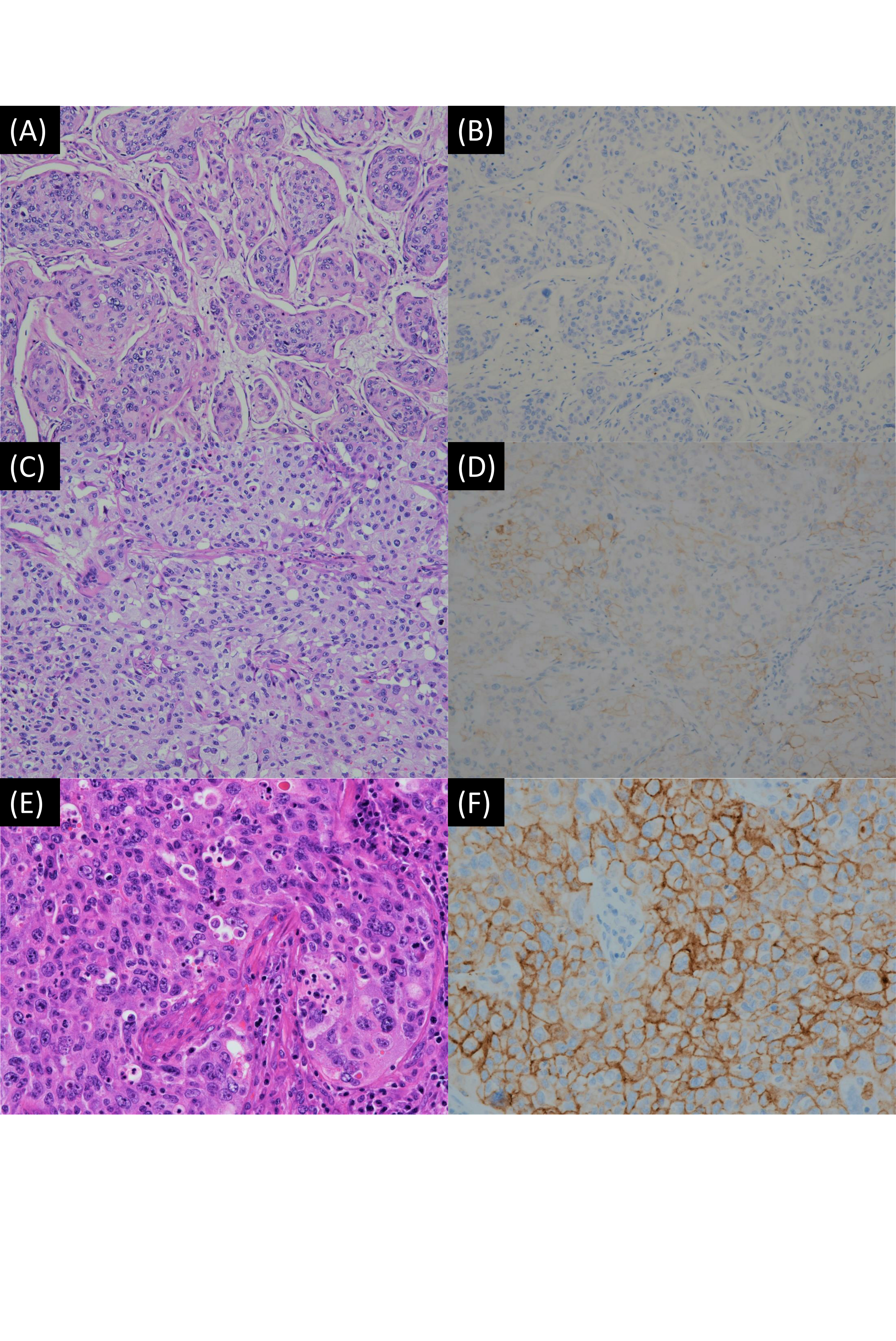

Supplement: Supplementary file 1 — Supplementary Figure S1. [file 41598_2022_7423_MOESM1_ESM.tif]

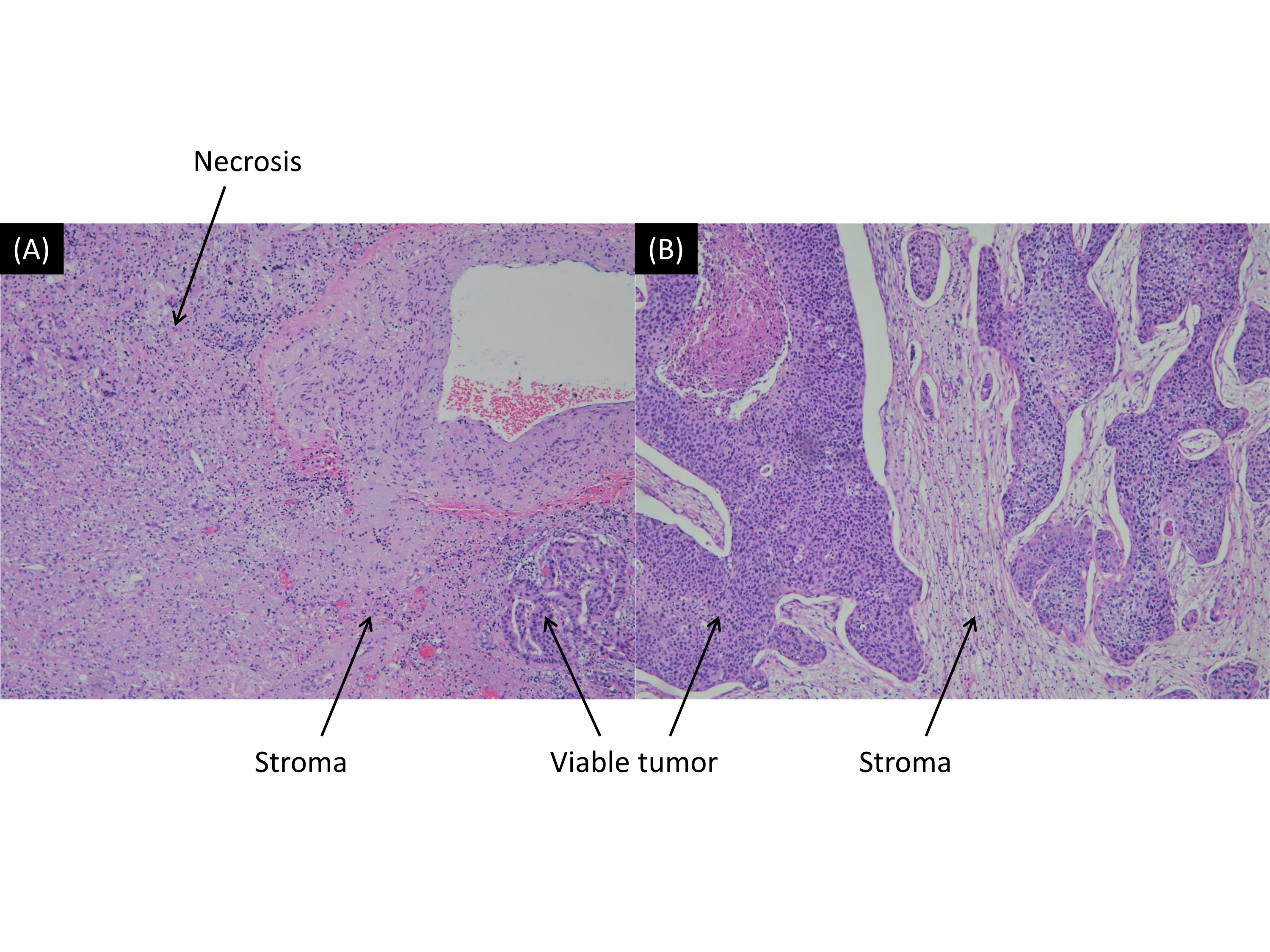

Supplement: Supplementary file 2 — Supplementary Figure S2. [file 41598_2022_7423_MOESM2_ESM.tif]

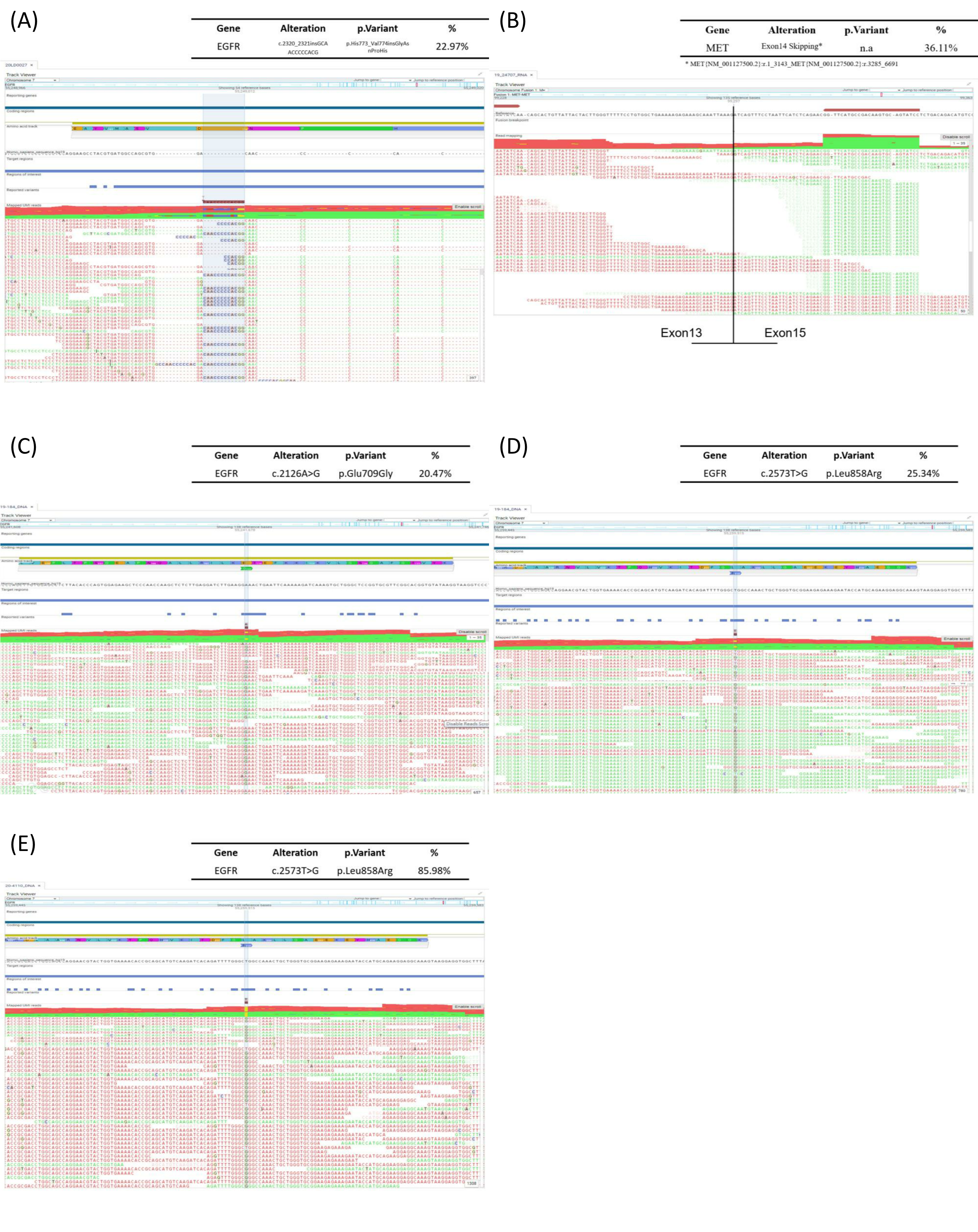

Supplement: Supplementary file 3 — Supplementary Figure S3. [file 41598_2022_7423_MOESM3_ESM.tif]
