# Supplementary material for: The impact of driver mutation on the treatment outcome of early-stage lung cancer patients receiving neoadjuvant immunotherapy and chemotherapy
Source: Sci Rep. 2022 Feb 28;12:3319. doi: 10.1038/s41598-022-07423-w (PMC8885645; doi:10.1038/s41598-022-07423-w)
Supplement: Supplementary file 4 — Supplementary Legends. [file 41598_2022_7423_MOESM4_ESM.docx]

**Supplementary figures**

Figure S1. Immunohistochemical staining of histopathologic pictures and associated PD-L1 expression in non-small cell lung cancer. (A,B) Tumor with negative PD-L1 expression (0%). (C,D) Tumor with low PD-L1 expression (1-49%). (E,F) Tumor with high PD-L1 expression (>50%).

Figure S2. The histopathologic pictures of resected lung cancer. (A) Tumor with major pathological response after neoadjuvant therapy. (B) Tumor without major pathological response after neoadjuvant therapy.

Figure S3. Genomic data from next generation sequencing of individual patients. Among three patients suffered from disease recurrence, all of them had targetable driver mutation, including one patient with EGFR exon 20 insertion (A), one patient with MET exon 14 skipping (B), and one patient with EGFR exon 18 E709K (C) and exon 21 L858R (D). Among 8 patients remaining in disease-free status, only one patient had targetable driver mutation (EGFR exon 21 L858R) (E).
